# Selective and hyperactive uptake of foreign DNA by adaptive immune systems of an archaeon via two distinct mechanisms

**DOI:** 10.1111/j.1365-2958.2012.08171.x

**Published:** 2012-07-27

**Authors:** Susanne Erdmann, Roger A Garrett

**Affiliations:** Archaea Centre, Department of Biology, University of CopenhagenOle Maaløes Vej 5, DK-2200 Copenhagen N, Denmark.

## Abstract

Central to the disparate adaptive immune systems of archaea and bacteria are clustered regularly interspaced short palindromic repeats (CRISPR). The spacer regions derive from invading genetic elements and, via RNA intermediates and associated proteins, target and cleave nucleic acids of the invader. Here we demonstrate the hyperactive uptake of hundreds of unique spacers within CRISPR loci associated with type I and IIIB immune systems of a hyperthermophilic archaeon. Infection with an environmental virus mixture resulted in the exclusive uptake of protospacers from a co-infecting putative conjugative plasmid. Spacer uptake occurred by two distinct mechanisms in only one of two CRISPR loci subfamilies present. In two loci, insertions, often multiple, occurred adjacent to the leader while in a third locus single spacers were incorporated throughout the array. Protospacer DNAs were excised from the invading genetic element immediately after CCN motifs, on either strand, with the secondary cut apparently produced by a ruler mechanism. Over a 10-week period, there was a gradual decrease in the number of wild-type cells present in the culture but the virus and putative conjugative plasmid were still propagating. The results underline the complex dynamics of CRISPR-based immune systems within a population infected with genetic elements.

## Introduction

Adaptive immune systems of most archaea and many bacteria primarily target invading viruses and conjugative plasmids and have recently been classified into major classes, denoted types I, II, IIIA and IIIB (Bolotin *et al*., 2005; Mojica *et al*., 2005; Pourcel *et al*., 2005; Makarova *et al*., 2011). Operationally, these CRISPR (clustered regularly interspaced palindromic repeats) systems share three main functional steps, adaptation, processing of CRISPR transcripts and interference, each associated with specific Cas proteins. Adaptation, the most conserved step mechanistically, involves the cleavage and uptake of foreign DNA as new spacers at, or near, the leader-adjacent repeat of CRISPR arrays (Barrangou *et al*., 2007; Deveau *et al*., 2008; Held and Whitaker, 2009; Lillestøl *et al*., 2009). Proteins Cas1, Cas2 and Cas4 have been implicated in this process, primarily on the basis of comparative genomic studies (reviewed in Garrett *et al*., 2011a; Makarova *et al*., 2011). CRISPR transcripts initiating within the leader region are cleaved specifically within repeat sequences to yield guide crRNAs either by the Cas6 protein for type I and III systems, or by a combination of a tracrRNA and RNase III in the bacteria-specific type II system (Tang *et al*., 2002; 2005; Brouns *et al*., 2008; Deltcheva *et al*., 2011). In the type IIIA and IIIB systems, targeting DNA and RNA respectively, guide crRNAs undergo further maturation at their 3′ ends (Hale *et al*., 2009; 2012; Hatoum-Aslan *et al*., 2011; Zhang *et al*., 2012). Functional interference complexes, consisting of guide crRNAs associated with a group of Cas proteins, target and cleave the nucleic acid components of invading genetic elements, and these modules generate most of the structural and functional diversity prevalent among CRISPR-based immune systems (Jore *et al*., 2011; Lintner *et al*., 2011; Zhang *et al*., 2012).

Although considerable progress has been made in elucidating details of the different CRISPR RNA processing and maturation mechanisms, and in determining the protein composition and structures of the different interference complexes involved in DNA and RNA targeting of type I and type IIIB systems, respectively, we still have limited insight into the nature and mechanism of the adaptation step. In early studies it was demonstrated that closely related strains of *Mycobacterium tuberculosis* carried different spacer sequences at one end of their CRISPR arrays, consistent with new spacers having been added (Hermans *et al*., 1991; Groenen *et al*., 1993) and this was later exploited as a typing strategy, spoligotyping (SPacer OLIGOnucleotide TYPING), where the variable region of the CRISPR array was characterized by PCR amplification and sequencing (Aranaz *et al*., 1996; Kamerbeek *et al*., 1997). Studies on archaeal CRISPR arrays, linked to the more complex CRISPR-based immune systems of different *Sulfolobus* species, underpinned this result by demonstrating the accumulation of multiple new spacers at the leader end of the CRISPR arrays but they also provided evidence for a complex picture of dynamic changes, including indels and rearrangements, occurring within the repeat arrays (Lillestøl *et al*., 2006; 2009; Held and Whitaker, 2009). Furthermore, environmental studies of CRISPR arrays of bacteria and archaea within biofilms provided evidence for a dynamic interplay between viruses and the spacer contents of CRISPR arrays. The results were consistent with mutations occurring in viral genomes to avoid targeting by guide crRNAs which resulted in the periodic uptake of new matching spacers at one end of the CRISPR arrays (Andersson and Banfield, 2008; Tyson and Banfield, 2008).

Successful uptake of spacers was first observed in the laboratory for the bacteria-specific type II CRISPR system of *Streptococcus thermophilus*. This process was induced by single phages and insertions occurred adjacent to leaders for two of three CRISPR loci which, in turn, led to phage resistance of the host. Single spacer inserts were detected in 39 phage-resistant mutants with a few carrying a further one to three spacer inserts (Barrangou *et al*., 2007; Deveau *et al*., 2008; Horvath *et al*., 2008). Very recently, unspecific uptake of chromosomal and plasmid vector protospacer DNA into CRISPR loci of *Escherichia coli* was shown to be induced by overexpression of two of the adaptation-associated proteins Cas1 and Cas2 (Yosef *et al*., 2012). Important for the adaptation mechanism are the short sequence PAM motifs (protospacer associated motif) located at the end of the protospacer that becomes leader proximal in the CRISPR array for all archaeal type I and III systems, and leader distal in the bacteria-specific type II system (Barrangou *et al*., 2007; Lillestøl *et al*., 2009; Mojica *et al*., 2009).

Here we investigated activation of the adaptation reaction in the model crenarchaeon *Sulfolobus solfataricus* P2 by viral infection. The organism is an excellent host for a variety of archaeal viruses and conjugative plasmids (Zillig *et al*., 1994; 1998). It carries six CRISPR loci, A to F, two gene cassettes encoding the adaptation-associated proteins Cas1, Cas2 and Cas4 (Garrett *et al*., 2011b) and three gene cassettes associated with type I and IIIB interference modules that target DNA and RNA respectively (Gudbergsdottir *et al*., 2011; Manica *et al*., 2011; Zhang *et al*., 2012). The six CRISPR arrays fall into two main crenarchaeal subfamilies on the basis of the sequences of their repeats, leaders, PAM motifs and associated Cas1 proteins (Lillestøl *et al*., 2009; Shah *et al*., 2009). Loci A and B belong to subfamily II and loci C to F belong to the more common subfamily I. Loci E and F carry repeats differing at one base pair from those of loci C and D and, whereas locus E contains a different type of leader, locus F has no leader and loci E and F are not physically proximal to adaptation-associated genes (Lillestøl *et al*., 2009). Infecting the *Sulfolobus* cells with a purified environmental virus mixture produced hyperactive adaptation of subfamily I CRISPR arrays C, D and E by two different mechanisms.

## Results

### Activation of adaptation by viruses

**Fig. 1:**
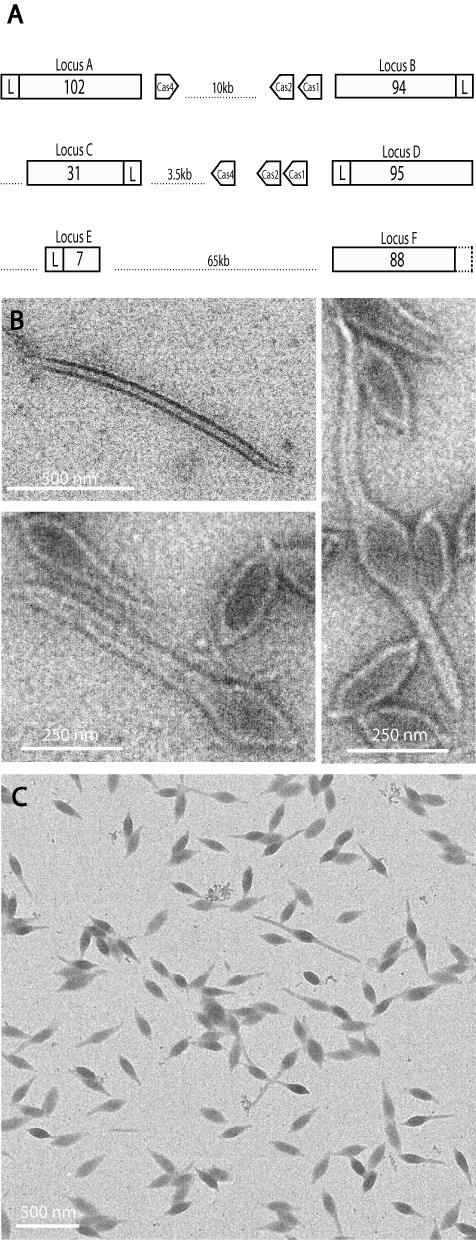
CRISPR loci and viruses infecting *S. solfataricus* P2. A. Scheme of six CRISPR loci of *S. solfataricus* P2, their associated leader regions (L), and genes encoding adaptation-associated Cas1, Cas2 and Cas4. Numbers of repeat-spacer units within CRISPR arrays are given. B and C. Electron micrographs of virus particles isolated from (B) supernatant of the enrichment culture, and (C) *S. solfataricus* P2 6 days post infection with the virus mixture in (B). Samples were negatively stained with 1% uranyl acetate and size bars are included.

Initial experiments were performed to induce new spacer uptake into the six CRISPR arrays A to F of *S. solfataricus* P2 (Fig. 1A) by infecting with single purified archaeal viruses, the rudivirus SIRV2, the bicaudavirus ATV and a tailed-fusiform virus STSV2. Only STSV2 (a variant of STSV1 –Xiang *et al*., 2005) propagated stably over longer periods but examination of the sizes of PCR products generated from the leader-proximal ends of the six CRISPR loci, tested over several weeks, failed to yield evidence for adaptation. Therefore, experiments were performed with an environmental sample containing archaeal viruses using the same PCR approach. An enrichment culture was established of a sample taken from a hot spring in Yellowstone National Park and virus-like particles were isolated from the supernatant. The main viral morphotypes present in the mixture were shown by electron microscopy to resemble closely those of crenarchaeal viruses, including single-tailed STSV1 and HAV2, two-tailed ATV and rod-shaped HAV1 (Fig. 1B) (Häring *et al*., 2005; Xiang *et al*., 2005; Garrett *et al*., 2010). This virus mixture was used to infect *S. solfataricus* P2. Examination of the viral content of the *S. solfataricus* P2-infected culture by electron microscopy 6 days post infection revealed that primarily the single-tailed fusiform virions were present with very few two-tailed virions and no rod-shaped virions (Fig. 1C).

**Fig. 2:**
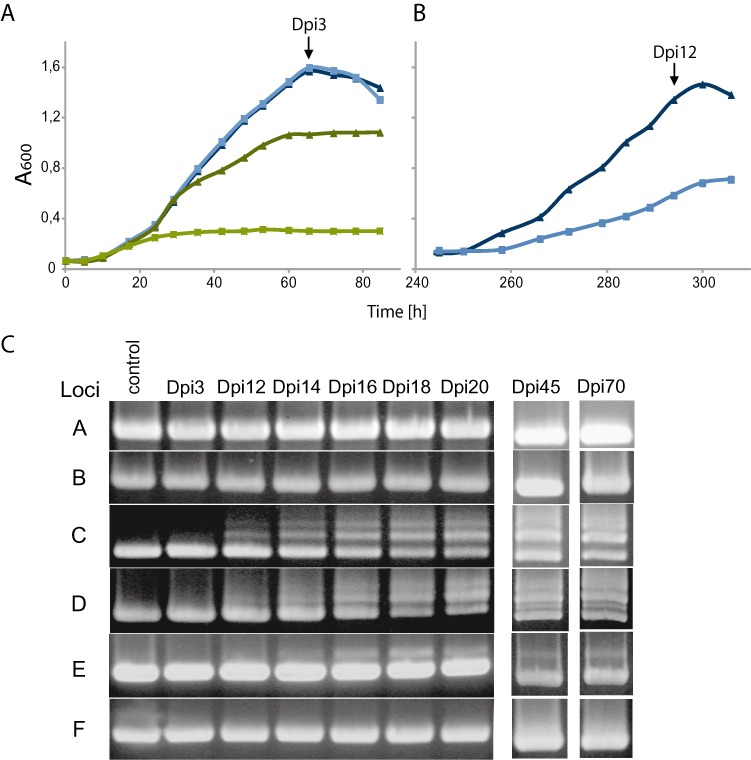
Growth curves of virus-infected *S. solfataricus* P2 and spacer uptake in CRISPR loci. A. Growth curves for uninfected (dark blue) and virus-infected (light blue) wild-type cells, and for uninfected (dark green) and infected (light green) CRISPR-minus strain. B. Growth curves for uninfected (dark blue) and virus-infected (light blue) wild-type cells at onset of spacer uptake (10–12 days). *A*_600_ measurements were made every 6 h. C. PCR products amplified from leader proximal regions of CRISPR loci A to F of wild-type strain and matched to growth curves (Dpi3 and Dpi12). Dpi, days post infection.

Initially, cultures of uninfected and virus-infected *S. solfataricus* P2 produced similar growth curves (Fig. 2A). In contrast a mutant P2 strain lacking CRISPR loci A to D and the adaptation-associated *cas* genes (Gudbergsdottir *et al*., 2011) showed retarded growth immediately after infection, consistent with a CRISPR-based defence operating only in the wild-type strain (Fig. 2A). Cultures were successively diluted to an *A*_600_ of 0.05 when stationary growth was reached every 3 days, with no addition of fresh virus mixture. Growth retardation of the infected wild-type cells was first observed 10 days post infection (Fig. 2B). This change preceded formation of larger amplified products from CRISPR loci indicative of the uptake of new spacers (Fig. 2C). Single larger fragments were observed after 12 days for loci C, D and E but not for loci A, B and F. Over 12–20 days, these larger bands increased in yield and, in addition, multiple bands appeared for loci C and D but not for locus E (Fig. 2C). When these experiments were repeated the onset of growth retardation varied in the range 8–20 days with detectable spacer uptake following 2–3 days later.

DNA from the larger PCR products was cloned and transformed into *E. coli*, and DNA from single colonies was sequenced through the leader proximal regions of loci C and D (five spacers each) and locus E (seven spacers). Surprisingly, large numbers of newly incorporated, unique, spacer sequences were found at each locus (Table 1). For loci C and D, 263 and 200 clones yielded 160 and 164 new unique spacers, respectively, and about 25% of the clones carried two to four new spacers. For locus E, 128 clones showed 94 unique single spacer inserts (Table 2). Moreover, a few identical spacers were taken up in different loci indicating that the adaptation process was not specific for a given CRISPR locus (Table 1). To screen for insertions or deletions within the whole of locus C, PCR analyses were performed throughout the locus (2 kb) and no changes were detected after 70 days of continuous culture.

**Table 1:** New spacers inserted into CRISPR loci C, D and E.

				Unique inserts of single spacers or unique combinations of spacers			
				
Locus	Total clones (expt. 1/2)	Total spacers	Unique spacers	Single	Two	Three	Four
C	263	333	160	124	39	5	0
D	200	260	164	126	39	3	1
E	128	128	94	94	0	0	0

Total numbers of clones sequenced for each locus are given. Some spacers unique for a given locus were shared: loci C + D (15 pairs), loci C + E (7 pairs), loci D + E (3 pairs) and loci C + D + E (1 triple) and a few spacers within multiple spacer inserts were identical to single spacer inserts.

**Table 2:** Distribution of new spacer inserts in CRISPR locus E where repeat 1 is leader proximal.

Locus E repeat number	Total clones	Unique spacers at a given repeat
1	18	16
2	1	1
3	13	13
4	85	53
6	10	10
7	1	1
Total	128	94

In total 128 independent clones were sequenced carrying a total of 94 different spacer sequences. Four pairs of spacers occurred at different repeats.

### Two different adaptation mechanisms

There is a major difference in the adaptation mechanisms of the different CRISPR loci. Loci C and D accrued one to four new spacers adjacent to the leader region, consistent with insertion occurring at the first repeat, although for clones exhibiting multiple new spacers the insertion order could not be ascertained. In contrast, only single spacer insertions were found for locus E and, moreover, they occurred at six of the eight repeats with a bias to repeat 4 (Table 2). These differences with respect to both the spacer insertion position and number of integrated spacers may reflect that locus E carries a different type of leader sequence from loci C and D (Gudbergsdottir *et al*., 2011). Moreover, the results indicate that for locus E, the repeats themselves are also important mechanistically for spacer insertion.

### Sequences of the virus mixture

DNA was extracted from the virus mixture that had been isolated from an infected culture of *S. solfataricus* P2 and purified on a CsCl gradient. The DNA was subjected to a round of Illumina sequencing with reads averaging about 90 bp. The sequences were assembled automatically using the CLC genomics workbench and the analysis yielded two sets of larger contigs one with a very high sequence coverage averaging 20 000-fold and another with a low coverage of five- to seven-fold. The contigs were analysed using Artemis (Rutherford *et al*., 2000) and several predicted ORFs for the high coverage element(s) showed best sequence matches mainly to ORFs of the bicaudavirus ATV and the tailed fusiform virus STSV1 (Häring *et al*., 2005; Xiang *et al*., 2005; Prangishvili *et al*., 2006b) summarized in Table 3. We inferred that these sequences derived from the dominant single-tailed virus that resembles STSV1 morphologically (Fig. 1C). Sequence alignments of these viral contigs with spacers of the six CRISPR loci of *S. solfataricus* P2 revealed in total eight perfect matches located in loci A (spacer 38) and D (spacers 24, 26, 34, 35, 37, 39 and 40 – all numbered from the leader) and specific TCN or CCN PAM motifs respectively. Moreover, spacers showing one to four mismatches which may have been active in interference (Gudbergsdottir *et al*., 2011; Manica *et al*., 2011) were present in loci B (spacer 34) and F (spacers 20, 21, 47, 59, 62 and 82).

**Table 3:** Assembled contigs of the high sequence coverage tailed fusiform virus.

			Best match			
					
Contig	Size (bp)	Orf size	Gene	Orf size	e value	Putative function
1	19325	588	ATV_66	618	1e_48	MoxR ATPase
		349	ATV_67	545	5e_05	Membrane protein
		222	ATV_35	220	0.006	–
		242	ATV_72	241	2e_128	Integrase
		105	ARV1_05	102	2e_13	–
		248	pNOB8_16	246	2e_134	–
		137	ATV_19	98	1e_23	–
		307	ATV_55	286	2e_28	Acetyl transferase
		319	ATV_34	330	3e_125	
2	10093	1642	STSV1_34	2308 Nter	4e_18	Structural
		126	ATV_42	145	4e_07	–
		279	ATV_56	277	6e_07	–
3	6753	143	Ahos_980	135	5e_53	GtrA family
		757	ATV_60	710	6e_97	Transmembrane
		153	ATV_62	131	3e_10	Coat protein
		373	ATV_71	1334 Cter	6e_23	Structural

Best sequence matches to the predicted contig ORFs are given together with expectancy values.

Analysis of the contigs with low sequence coverage showed that most predicted ORFs yielded best matches to ORFs of conjugative plasmids of the Sulfolobales with the *Acidianus* plasmid pAH1 and the *Sulfolobus* plasmid pARN3 dominating (Table 4) (Greve *et al*., 2004; Basta *et al*., 2009). In addition, there were a few best matches to putative conjugative plasmid regions integrated within chromosomes of members of the Sulfolobales. In contrast to the results for the virus, no significant sequence matches were observed between the contigs of the putative conjugative plasmid and the spacers of the six CRISPR loci of *S. solfataricus* P2. The best match was to spacer 49 of locus A with six mismatching nucleotides.

**Table 4:** Contigs assembled for the low sequence coverage putative conjugative plasmid.

Contig	Size (bp)	Matching spacers	Unique spacers	Forward/reverse	ORFs	Orf match	e value	Putative function
1	7277	151	125	60/65	200	pARN3_06	7e_62	Conserved plasmid
					472	pARN3_05	0.0	Membrane protein
					622	pAH1_04	0.0	TrbE-like
					223	pARN3_02	2e_85	Conserved plasmid
					712	pARN3_01	0.0	Conserved plasmid
2	6723	141	123	66/57	192	pAH1_09	1e_50	Membrane protein
					182	pAH1_p11	2e_93	Membrane protein
					132	pARN3_10	2e_55	Conserved plasmid
					1029	pAH1_p13	0.0	TraG-like
3	2469	63	50	21/29	95 (Nter.)	pNOB8_19	2e_33	Conserved plasmid
					93	pNOB8_18	2e_09	Conserved plasmid
					196	pARN3_21	2e_45	Conserved plasmid
					174	pSOG1_16	1e_11	DNA binding
4	2003	20	19	12/7	95	pAH1_31	1e_54	Conserved plasmid
					91	pKEF9_29	2e_29	Conserved plasmid
					71	pSOG1_27	1e_21	Conserved plasmid
					94	pNOB8_01	0.030	Conserved plasmid
					158	pAH1_23	1e_63	Conserved plasmid
5	1780	31	25	17/8	435	pHVE14_20,21	0.0	OrfA/B IS200/605
6	1426	17	16	8/8	83	pHVE14_41	8e_44	PlrA protein
					228	*M. sedula* Msed_2202	3e_161	Unnamed protein
7	1314	14	11	7/4	361 (Cter.)	pKEF9_31	0.0	Integrase
8	412	10	7	4/3	102	pARN3_15	6e_22	Conserved plasmid
9	185	9	8	5/3	partial	*A. hospitalis* Ahos_0414	2e_84	Transporter Orf393
10	152	23	21	12/9	partial	Ahos_0414	8e_18	Transporter Orf393

The sizes are given together with the number and directions of the matching spacers. The best sequence matches to the predicted contig ORFs are given together with their expectancy values.

The presence of a putative conjugative plasmid in the virus preparation was unexpected given that the virus mixture was purified by buoyant density gradient centrifugation and that the plasmid was not detectable by PCR in the wild-type *S. solfataricus* P2 strain prior to infection with the virus mixture. To test for its presence in the purified virus mixture, the latter was treated with DNase I before extracting the DNA from the virus particles. A decrease in DNA concentration was detected, probably due to virus particles having been disrupted during the purification process, but PCR amplification of the *traG*-like gene after DNA digestion confirmed the presence of the putative conjugative plasmid in the virus mixture (Fig. S1).

### Location and distribution of protospacer sites

Sequences of new spacer inserts were assembled automatically with all the larger contigs deriving from the virus mixture (Tables 3 and 4) and, to our surprise, they assembled exclusively on the low coverage contigs of the putative conjugative plasmid. The number of spacers associated with each contig are listed in Table 4. The spacers were distributed fairly evenly along each of the DNA contigs and they were arranged almost equally on the two strands (Table 4), consistent with earlier bioinformatic analyses (Shah *et al*., 2009; 2011). Only 32 of the 418 sequenced spacers could not be directly matched to the contigs and we assume they lie within gaps in the conjugative plasmid sequence.

### CCN PAM motif was invariant for CRISPR loci C, D and E

**Fig. 3:**
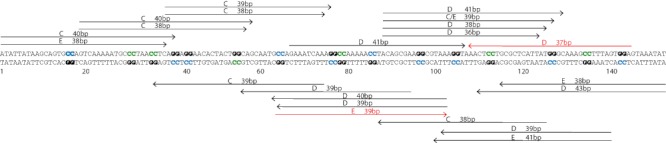
Assembly of newly inserted spacer sequences. Composition of one contig generated from overlapping spacer sequences on both DNA strands. Arrowheads indicate the direction away from the leader (corresponding to spacer crRNA sequence). Two spacers (in red) have opposite orientations. Spacers are preceded by a CCN protospacer-associated motif (PAM) (Lillestøl *et al*., 2009) (in blue and bold type). CCN motifs with no detected spacers (green). Loci origins and spacer lengths are indicated.

Although the protospacers are distributed fairly evenly along the contigs there were a few local clusters of spacer matches. These were detected initially when we attempted to assemble the newly incorporated spacer sequences alone. They yielded a few small contigs of 80–150 bp each carrying multiple unique spacer sequences, exemplified by the 152 bp contig in Fig. 3. These provided insights into both the specificity and the variety of protospacer excision sites. The leader proximal end of the spacer sequence was adjacent to an invariant CCN protospacer adjacent motif (PAM), located on either DNA strand as predicted earlier for crenarchaeal subfamily I CRISPR arrays (Lillestøl *et al*., 2009; Mojica *et al*., 2009; Shah *et al*., 2009). For overlapping, near identical spacers, the PAM-associated ends were homogeneous with heterogeneities occurring only when preceded by overlapping PAM motifs including -CCC- or -CCCC-. In contrast, leader distal ends were heterogeneous with no evidence of any base or sequence specificity at the cleavage site (Fig. 3). Given that spacer lengths varied from 36 to 43 bp (Fig. 3), it is inferred that the cleavage occurs by an imprecise ruler mechanism measured from the PAM motif site. Rare examples of apparent inverted spacer insertion were detected, relative to the PAM motif site, and two are colour-coded in Fig. 3. This general pattern of protospacer cleavage was conserved throughout the contigs of the putative conjugative plasmid (Table 4).

### Simultaneous intracellular adaptation of three CRISPR loci and their effect on genetic elements

Single clones were generated from the culture approximately 15 days after the onset of adaptation (day 27). Fourteen clones were selected that showed PCR evidence for new spacer uptake in the CRISPR loci C, D and E. Sequencing revealed that each of the clones carried new spacer inserts. Six clones contained new spacers in either locus C or D, seven clones had acquired spacers in both loci C and D, and one clone exhibited new spacers in all three loci (Table 5). One-third of the new inserts in loci C and D were of two or three spacers. On balance locus D appeared more active than locus C carrying 21 versus 13 new spacers and this may correlate with locus D being much larger than locus C in the wild-type strain (Fig. 1A). Locus E exhibited a lower activity level.

**Table 5:** Sequences of newly integrated spacers identified in 14 single clones (S1 to S14) generated 15 days after the onset of adaptation (day 27)

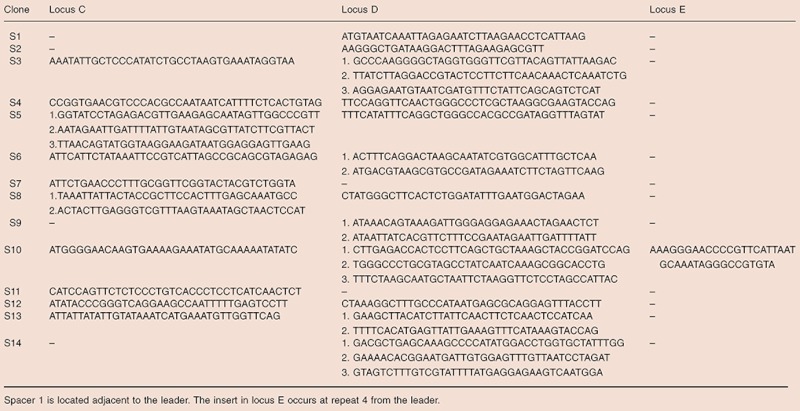

Three of the single clones, S11 with an insertion in locus C, S3 with an insertion in loci C and D and S10 with insertion in loci C, D and E (Table 5), and an additional purified clone R1 devoid of additional spacers were cultured, with the wild-type strain as control, and reinfected with the purified virus mixture. Growth rates of each of the four single clones did not differ significantly from those of the wild-type control and no new spacer uptake was detected 25 days post infection.

**Fig. 4:**
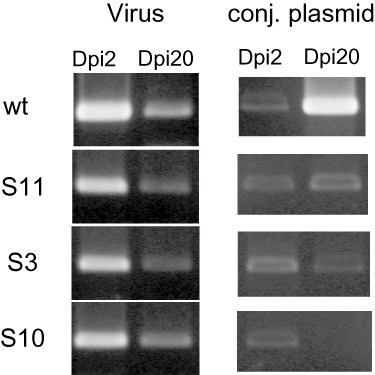
Yields of virus and putative conjugative plasmid in single clones carrying new spacers. Reinfection with the purified virus mixture of isolated single clones containing new spacers in locus C (S11), loci C and D (S3) and in loci C, D and E (S10). PCR products of *orf174* within an operon in viral contig 1 (Table 3) and of the *traG*-like gene in contig 2 of the putative conjugative plasmid (Table 4) were PCR-amplified from DNA isolated from cultures 2 and 20 days post infection (Dpi).

PCR amplification of *orf174* within an operon in viral contig 1 (Table 3) showed that each of the three clones S3, S10 and S11, as well as R1, could be reinfected with the tailed fusiform virus (Fig. 4). Amplification of the *traG*-like gene located in contig 2 of the putative conjugative plasmid by PCR (Table 4), showed that the plasmid was not detectable after about 20 days post infection in cultures of the single clones S3, S10 and S11 as a result of being out-diluted by successive dilutions every 3 days. However it was propagating in the wild-type culture (Fig. 4). In contrast, the single clone R1 was still sensitive to the putative conjugative plasmid but yields were constantly lower than for the wild-type strain over a 30-day period (data not shown). Thus, only the single clones with newly acquired spacers were resistant to the putative conjugative plasmid and we infer, therefore, that the latter was confined to cells for which adaptation had not been activated in the infected wild-type culture.

### Challenging a CRISPR-minus mutant with the virus mixture

Growth curves demonstrated that the mutant which lacked CRISPR loci A to D and the adaptation-associated genes showed retarded growth immediately after infection with the purified virus mixture (Fig. 2A). However, in two out of three cultures the growth recovered and, after several days of culturing, CRISPR loci A to D were detected in the culture (Fig. S2), implying that a small residual population of CRISPR loci-containing cells had survived in the original mutant (Gudbergsdottir *et al*., 2011). After 12 days multiple PCR bands were generated from the cells. The bands were cloned and sequenced and revealed the presence of new spacers in each of loci C, D and E, many of which were identical in sequence and all matches were to the putative conjugative plasmid. Of 55 clones, 29 yielded one unique spacer sequence, 10 produced another, and in total there were only 17 unique sequences. This contrasts with the high percentage of unique spacers observed in the infected wild-type culture (Tables 1 and 2). We infer that adaptation occurred in the small percentage of cells carrying CRISPR loci A to D and the adaptation-associated proteins (Fig. 1A), and that these then rapidly outgrew the CRISPR-minus cells and dominated the culture.

### Longer-term viability of the genetic elements

**Fig. 5:**
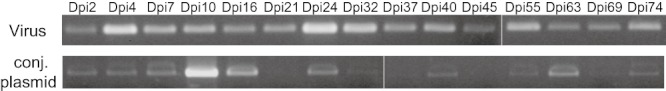
Yields of virus and putative conjugative plasmid in the wild-type culture over a 74-day period. PCR products are shown for *orf174* of the virus and the *traG*-like gene of the putative conjugative plasmid isolated from cultures 2–74 days post infection (Dpi). Template DNA was isolated from 2 ml of infected cells and concentrations were adjusted to be the same for each sample prior to PCR analysis.

The infected wild-type culture was continued beyond 74 days, with successive dilutions every 3 days at stationary phase. The growth rate recovered after about 35 days and PCR amplification of CRISPR loci C to E, after 45 days and 70 days, showed no substantial changes in the intensity ratio between the wild-type band and the larger PCR products carrying new spacer inserts (Fig. 2C). Single clones examined after culturing for 30 days (14 with insertions and 24 without), after 37 days (13 with insertions and 6 without) and after 70 days (23 with insertions and 7 without) revealed a progressive increase in the percentage of clones carrying new spacer inserts in any CRISPR locus. Moreover, PCR products obtained under the same conditions from culture samples taken at regular intervals throughout the 74-day period showed that the level of viral and plasmid DNA oscillated, although the level of plasmid DNA was generally very low (Fig. 5). The presence of the virus after 70 days was also confirmed by electron microscopy (data not shown).

## Discussion

Archaeal viruses are extremely diverse both morphologically and in their genomic properties and they have been classified into several new viral families, distinct from those of bacteria and eukarya (Prangishvili *et al*., 2006a; Porter *et al*., 2007; Pina *et al*., 2011). A distinguishing property of many of the viruses, and especially those found in high-temperature environments is that they propagate in carrier-like states with their hosts, often together with other viruses, and very few have been shown to induce cell lysis (Prangishvili *et al*., 2006a; Pina *et al*., 2011). This may reflect a tendency to minimize contact with harsh extracellular conditions. Moreover, it has been suggested that CRISPR-based immune systems, which are often complex and disparate in hyperthermophilic archaea, may play a regulatory role in maintaining viruses in low copy numbers (Lillestøl *et al*., 2009; Garrett *et al*., 2011a, b).

Our initial attempts to induce CRISPR adaptation by infecting *S. solfataricus* P2 singly with the rudivirus SIRV2, the bicaudavirus ATV and the tailed-fusiform virus STSV2 were unsuccessful which suggested that activation of the adaptation mechanism might be more complex in *Sulfolobus* than in the bacteria-specific system of *S. thermophilus* where single phage infections induced adaptation (Barrangou *et al*., 2007; Deveau *et al*., 2008). Successful uptake of spacers could only be induced when employing an environmental sample containing viruses and a putative conjugative plasmid. Selective targeting of the low level putative conjugative plasmid but not of the viral DNA was unexpected given the 1:3300 DNA weight ratio estimated from sequence coverage. Nevertheless, previous analyses of *Sulfolobus* CRISPR loci identified many spacers predicted to arise from conjugative plasmids (Lillestøl *et al*., 2006; Shah *et al*., 2009) and this may reflect that these plasmids can propagate and spread rapidly and efficiently in *Sulfolobus* cultures if unchallenged (Prangishvili *et al*., 1998; Zillig *et al*., 1998). Co-migration of the putative plasmid with virions in bouyant density gradients, and its resistance to DNase I treatment, suggested that it may have been encapsulated by viral coat proteins and transferred as a virus satellite, as has been observed for cryptic plasmids of *Sulfolobus* (Arnold *et al*., 1999; Wang *et al*., 2007). Another possibility is that it was extruded from *S. solfataricus* cells within vesicle particles (Soler *et al*., 2008).

Non-targeting of the virus by the activated adaptation mechanism of the host was not anticipated although it is consistent with many crenarchaeal viruses coexisting in stable carrier relationships with their hosts (Prangishvili *et al*., 2006a). However, this observation became more intriguing when we discovered that the viral contigs carried eight perfect sequence matches to spacers within loci A (one) and D (seven) as well as specific PAM motifs. This was surprising first because the host was isolated from Pisciarelli near Naples whereas the viruses were sampled in Yellowstone National Park, USA, and it suggested that the virus and or host are more mobile geographically than previously imagined. Second, of the three single viruses employed unsuccessfully in preliminary adaptation studies, SIRV2 and ATV infected the CRISPR-minus mutant but did not propagate in wild-type *S. solfataricus* P2 which carries perfect spacer matches against each virus (Shah *et al*., 2009; Gudbergsdottir *et al*., 2011). In contrast STSV2, carrying no significant spacer matches, propagated in wild-type *S. solfataricus* P2 but did not induce adaptation (S.E. and R.A.G., unpublished). However, exceptionally for crenarchaeal viruses, SIRV2 and ATV, in contrast to STSV2 and the Yellowstone virus, have been shown to induce cell lysis (Prangishvili *et al*., 2006b; Bize *et al*., 2009). Possibly, they elicit a strong response from the CRISPR immune system at the onset of lysis. Nevertheless, it is evident that the tailed-fusiform virus described here is not extinguished by the wild-type *S. solfataricus* P2 CRISPR interference response (Gudbergsdottir *et al*., 2011; Zhang *et al*., 2012), nor is it susceptible to the host adaptation apparatus despite the latter having been activated. At present we do not understand the mechanism underlying the resistance but it is unlikely that the virus interferes with expression of the adaptation gene cassette by integration in a *csa3* gene encoding a putative transcriptional regulator, as has been inferred for another *Sulfolobus* virus (Shah *et al*., 2011), because this would have blocked all uptake of protospacers, although it could have played a role in specifically blocking adaptation of CRISPR loci A and B.

Induction of spacer uptake in *Sulfolobus* required infection by a virus mixture and was highly specific for (i) the *Sulfolobus* subfamily I CRISPR arrays and (ii) a single genetic element in the mixture; moreover, no spacers derived from the *S. solfataricus* P2 chromosome (She *et al*., 2001). This contrasts with the recent demonstration that CRISPR adaptation in *E. coli*, induced by overexpression of adaptation-associated Cas1 and Cas2, was relatively unspecific in acquiring many spacers from the host chromosome although the targeting of chromosomal DNA could reflect the absence of an active interference system in these strains (Yosef *et al*., 2012). The regulation of adaptation in *Sulfolobus* is likely to be complex but possibly the third adaptation-related protein Cas4, which is generally encoded in gene cassettes together with Cas1 and Cas2 in archaea (Shah and Garrett, 2011), facilitates specific recognition and fragmentation of invading genetic elements.

Wild-type *S. solfataricus* appeared resistant to the virus mixture with no reduction in growth over the first 10 days post infection, in contrast to the CRISPR-minus mutant. Subsequently growth was retarded and this coincided, 12 days post infection, with CRISPR loci C, D and E rapidly acquiring hundreds, and probably thousands, of new spacers, often in multiple unique copies per CRISPR locus, and in multiple CRISPR loci for a given cell. In total, we sequenced about 420 unique spacers deriving from the putative conjugative plasmid. Given that the CCN PAM motifs occur on average every 10 bp in the contigs (see Fig. 3), this probably constitutes a small proportion of those newly acquired spacers present in the population. Diversity of the protospacer sequences is also enhanced by the variable cleavage distance, 39 (± 4) nucleotides, from the PAM motif although this sequence heterogeneity probably produces minimal benefits for the effectivity of the CRISPR system, a supposition that is strengthened by the observation that overlapping spacers rarely occur within CRISPR loci found in natural *Sulfolobus* strains (Lillestøl *et al*., 2006; 2009; Shah *et al*., 2009).

Loci A and B were both inert to adaptation despite their actively taking up new spacers in closely related *S. solfataricus* strains (Lillestøl *et al*., 2006; 2009) and the observation that their transcripts are constitutively expressed and processed *in vivo* (Lillestøl *et al*., 2009; Wurtzel *et al*., 2010; Deng *et al*., 2012). However, their assignment to the less common subfamily II crenarchaeal CRISPR loci, on the basis of the sequences of their leaders, repeats, adaptation-linked Cas1 proteins and differing TCN PAM motif (Lillestøl *et al*., 2009; Gudbergsdottir *et al*., 2011), suggests that their adaptation mechanism may be activated differently. We infer that the inactivity of locus F in new spacer uptake is due to its lacking a leader region (Lillestøl *et al*., 2006).

The adaptation mechanism for loci C and D follows the pattern seen for the bacteria-specific type II CRISPR system of *S. thermophilus* and very recently in the type IE system of *E. coli* where new spacers were added adjacent to the leader on phage infection (Barrangou *et al*., 2007; Deveau *et al*., 2008; Horvath *et al*., 2008; Yosef *et al*., 2012). Clearly, the spacer insertion mechanism operating on locus E of *Sulfolobus* differs both with respect to position, since few insertions occur at repeat 1 (Table 2), and in being limited to single spacer insertions. The result also undermines the consensus hypothesis that spacer order in CRISPR arrays invariably provides a chronological record of genetic element invasion. The altered mechanism may reflect that locus E is associated with a different type of leader sequence common to *Sulfolobus islandicus* species, from which it may derive (Garrett *et al*., 2011b), and it requires complementation by Cas proteins associated with other CRISPR loci, probably loci C and D carrying similar repeats (Lillestøl *et al*., 2009). These properties may alter the compatibility between the Cas proteins and the leader of locus E leading to a less stringent insertion mechanism. Although there is support for the leader playing an important functional role (Marraffini and Sontheimer, 2008; Lillestøl *et al*., 2009; Yosef *et al*., 2012), possibly as an assembly site for the adaptation-associated Cas proteins, for locus E the repeats themselves appear to be important mechanistic determinants of adaptation.

After this article was submitted, a second report of spacer uptake in type IE CRISPR systems of *E. coli* strains appeared, in which it was claimed that the 3′-nucleotide of the AAG PAM motif became part of the first repeat during adaptation (Swarts *et al*., 2012). For the *Sulfolobus* systems described here, the third nucleotide of the CCN PAM motif is not conserved and therefore the same mechanism cannot apply.

In the long-term infection experiments, continued beyond 74 days, we observed an increase in the proportion of cells carrying spacer insertions, but neither the wild-type host nor the invading genetic elements were extinguished from the culture. Thus, virus–plasmid–host conflicts are ongoing and long term. Presumably when cells are no longer disadvantaged by mutated elements there must be a very strong selection for a limited number of spacers that are especially effective, possibly corresponding to functionally critical, and relatively conserved, genomic sites such as origins of replication.

In conclusion, we have developed a natural system to induce new spacer uptake in a subfamily of CRISPR loci of the model crenarchaeon *Sulfolobus* which allows us to study, in detail, the molecular mechanisms involved in the adaptation process, the molecular basis for viral resistance to the CRISPR-based immune systems, and the longer-term selection processes occurring within the CRISPR-based immune systems of infected *Sulfolobus* populations.

## Experimental procedures

### Isolation of virus particles and infection of *S. solfataricus* P2

An aqueous mud sample was taken from an acidic hot spring (pH 2, 85°C) in Monument Geysir Basin, Yellowstone National Park. One millilitre of this sample was added to 50 ml of *Sulfolobus* medium supplemented with 0.2% trypton, 0.1% yeast extract and 0.2% sucrose (TYS medium) (Zillig *et al*., 1994) and incubated aerobically for 5 days at 78°C. Two litres of enrichment culture was then established in TYS medium at 78°C. Cells were pelleted (6000 *g*, 10 min) and virus particles were isolated by filtration of the supernatant through 0.2 µm pore filters (Nalgene®). This virus mixture was then used to infect *S. solfataricus* P2 (DSM 1617) cultured in the *Sulfolobus* medium (Zillig *et al*., 1994).

Cells of *S. solfataricus* P2 were harvested from fresh culture by centrifuging (6000 *g*, 10 min) and resuspending in 1 ml of TYS medium. Twenty microlitres of virus mixture was added at 3 PFU µl^−1^ and after incubating for 2 h at 80°C, infected cells were transferred to 50 ml of pre-heated (78°C) TYS medium. Infected *S. solfataricus* P2 was then incubated at 78°C for 3–6 days before isolating infectious particles as described above for the enrichment culture. Infections with the single viruses SIRV2, ATV and STSV2 were performed using the same procedure.

### Sequencing of the virus mixture

*Sulfolobus solfataricus* cells were infected and grown for 3 days in 2 l of TYS medium. Cells were separated by centrifugation (6000 *g*, 10 min) and virus particles were isolated from the supernatant by filtration using Vivaspin 20 columns (1 000 000 Mwco, Sartorius Stedim, Aubagne, France) at 1500 *g* and dissolved in 10 mM Tris-HCl, pH 8.0. The virus mixture was then loaded onto 0.45 g ml^−1^ CsCl and centrifuged for 48 h at 38 000 r.p.m. in a SW41 rotor (Beckman, Fullerton, USA). The virus band was extracted from gradients and CsCl was removed by dialysis against 10 mM Tris-HCl, pH 8. DNA was isolated using DNeasy® Blood&Tissue Kit (Qiagen, Hilden, Germany). Sequencing by Illumina sequencing and raw data treatment was performed by Beijing Genomics Institute (Shenzhen, China). Clean data consisting of approximately 90 bp DNA fragments were assembled using the CLC genomics workbench (CLC Bio, Aarhus, Denmark). *orf174* within an operon in viral contig 1 and the *traG*-like gene in contig 2 of the putative conjugative plasmid were selected to detect the appropriate genetic element in host cells. DNA was amplified by PCR using forward and reverse primers 5′-CCCACCTATATCGAATTC-3′ and 5′-GTGTCTCTCATATTTGCAATC-3′, respectively, for the virus and 5′-GCCTTAGCGAGGGCCCAGTTGAACCTGG-3′ and 5′- CTATCCTATCCCTGTCTATCCCTAG-3′, respectively, for the putative conjugative plasmid. DNA from the initial enrichment culture and the *S. solfataricus* P2 culture were used as positive and negative controls respectively.

### DNase I treatment of the virus mixture

The virus mixture obtained from infected *S. solfataricus* cells was purified in a CsCl density gradient followed by dialysis against 10 mM Tris-HCl, pH 8. One hundred and fifty microlitres of purified virus mixture was digested with DNase I (Fermentas, St. Leon-Rot, Germany) at 37°C. DNA of the digested virus mixture and an undigested 150 µl sample was isolated using DNeasy® Blood&Tissue Kit (Qiagen). DNA concentration was measured using NanoDrop®.

### Electron microscopy

Virus particles were adsorbed onto carbon-coated copper grids for 5 min and stained with 2% uranyl acetate. Images were recorded using a Tecnai G2 transmission electron microscope (FEI, Eindhoven, Netherlands), with a CCD camera, at an acceleration voltage of 120 kV.

### Growth curves and PCR amplification of leader proximal CRISPR regions

Wild-type P2 strain and the mutant strain lacking CRISPR loci A to D and their associated *cas* genes, were infected with virus mixture and cultivated, with an uninfected control sample, in 50 ml of TYS medium at 78°C. One millilitre of samples of each culture was taken every 6 h and optical densities were recorded at 600 nm. Two millilitres of samples of each culture was taken every 24 h and cells were harvested by centrifugation (6000 *g*, 10 min). DNA was isolated using DNeasy® Blood&Tissue Kit (Qiagen). The leader proximal regions of the six CRISPR loci were amplified by PCR using forward primers 5′-AGCTTCTGACCCGCTCCTGA-3′ for locus A, 5′-AGGGGTTTGTGGGATGGGTTGTG-3′, for locus B, 5′-TCGCTTATCTCTCTCATGCGCCATT-3′ for locus C, 5′-AGTTCCACCCCCGAAGCTCCT-3′ for locus D, 5′-ATAGGGAAAGAGTTCCCCCG-3′ for locus E, 5′-CGGCGTTATAATGGGTATCGGAATCGG-3′ for locus F and reverse primers 5′-GCACATCATCAAACAATGGTAAGCC-3′ for locus A, 5′-ACAACTACCACCACTACCACGG-3′ for locus B, 5′-TGTCCCGTTTTTGTAAGTGGGGG-3′ for locus C, 5′-AGCCGGGACAAGTTTCACAAATTGA-3′ for locus D, 5′-TGACTCTAGTGCAATCTTCGA-3′ for locus E, 5′-GCTCACTATCTCACCCCTATCAATACCC-3′ for locus F. Single clones of infected *S. solfataricus* cells were obtained on Gel-rite plates and cultured in TYS medium (Zillig *et al*., 1994).

### Cloning of PCR products and sequencing

PCR products were separated on 1.5% agarose gels and bands larger than the those of the uninfected control sample were excised from the gel and purified with QIAquick Gel Extraction Kit (Qiagen). Purified PCR products were cloned using InsTAclone™PCR Cloning Kit (Fermentas) following the manufacturers' protocols. Plasmid purification and sequencing were performed by GATC Biotech AG, (Konstanz, Germany).

## References

[b1] Andersson AF, Banfield JF (2008). Virus population dynamics and acquired virus resistance in natural microbial communities. Science.

[b2] Aranaz A, Liébana E, Mateos A, Dominguez L, Vidal D, Domingo M (1996). Spacer oligonucleotide typing of *Mycobacterium bovis* strains from cattle and other animals: a tool for studying epidemiology of tuberculosis. J Clin Microbiol.

[b3] Arnold HP, She Q, Phan H, Stedman K, Prangishvili D, Holz I (1999). The genetic element pSSVx of the extremely thermophilkic crenarchaeon *Sulfolobus* is a hybrid between a plasmid and a virus. Mol Microbiol.

[b4] Barrangou R, Fremaux C, Deveau H, Richards M, Boyaval P, Moineau S (2007). CRISPR provides acquired resistance against viruses in prokaryotes. Science.

[b5] Basta T, Smyth J, Forterre P, Prangishvili D, Peng X (2009). Novel archaeal plasmid pAH1 and its interactions with the lipothrixvirus AFV1. Mol Microbiol.

[b6] Bize A, Karlsson EA, Ekefjard K, Quax TE, Pina M, Prevost MC (2009). A unique virus release mechanism in the Archaea. Proc Natl Acad Sci USA.

[b7] Bolotin A, Quinquis B, Sorokin A, Ehrlich SD (2005). Clustered regularly interspaced short palindrome repeats (CRISPRs) have spacers of extrachromosomal origin. Microbiology.

[b8] Brouns SJ, Jore MM, Lundgren M, Westra ER, Slijkhuis RJ, Snijders AP (2008). Small CRISPR RNAs guide antiviral defense in prokaryotes. Science.

[b9] Deltcheva E, Chylinski K, Sharma CM, Gonzales K, Chao Y, Pirzada ZA (2011). CRISPR RNA maturation by trans-encoded small RNA and host factor RNase III. Nature.

[b10] Deng L, Kenchappa CS, Peng X, She Q, Garrett RA (2012). Modulation of CRISPR locus transcription by the repeat-binding protein Cbp1 in *Sulfolobus*. Nucleic Acids Res.

[b11] Deveau H, Barrangou R, Garneau JE, Labonté J, Fremaux C, Boyaval P (2008). Phage response to CRISPR-encoded resistance in *Streptococcus thermophilus*. J Bacteriol.

[b12] Garrett RA, Bize A, Shah SA, Stetter K, Prangishvili D, Peng X (2010). Metagenomic analyses of novel viruses, plasmids, and their variants, from an environmental sample of hyperthermophilic neutrophiles cultured in a bioreactor. Environ Microbiol.

[b13] Garrett RA, Vestergaard G, Shah SA (2011a). Archaeal CRISPR-based immune systems: exchangeable functional modules. Trends Microbiol.

[b14] Garrett RA, Shah SA, Vestergaard G, Deng L, Gudbergsdottir S, Kenchappa CS (2011b). CRISPR-based immune systems of the Sulfolobales – complexity and diversity. Biochem Soc Trans.

[b15] Greve B, Jensen S, Brügger K, Zillig W, Garrett RA (2004). Genomic comparison of archaeal conjugative plasmids from *Sulfolobus*. Archaea.

[b16] Groenen PM, Bunschoten AE, van Soolingen D, van Embden JD (1993). Nature of DNA polymorphism in the direct repeat cluster of *Mycobacterium tuberculosis*; application for strain differentiation by a novel typing method. Mol Microbiol.

[b17] Gudbergsdottir S, Deng L, Chen Z, Jensen JVK, Jensen LR, She Q (2011). Dynamic properties of the *Sulfolobus* CRISPR/Cas and CRISPR/Cmr systems when challenged with vector-borne viral and plasmid genes and protospacers. Mol Microbiol.

[b18] Hale CR, Zhao P, Olson S, Duff MO, Graveley BR, Wells L (2009). RNA-guided RNA cleavage by a CRISPR RNA–Cas protein complex. Cell.

[b19] Hale CR, Majumdar S, Elmore J, Pfister N, Compton M, Olson S (2012). Essential features and rational design of CRISPR RNAs that function with the Cas RAMP module complex to cleave RNAs. Mol Cell.

[b20] Häring M, Vestergaard G, Rachel R, Chen L, Garrett RA, Prangishvili D (2005). Independent virus development outside a host. Nature.

[b21] Hatoum-Aslan A, Maniv I, Marraffini LA (2011). Mature clustered, regularly interspaced, short palindromic repeats RNA (crRNA) length is measured by a ruler mechanism anchored at the precursor processing site. Proc Natl Acad Sci USA.

[b22] Held NL, Whitaker RJ (2009). Viral biogeography revealed by signatures in *Sulfolobus islandicus* genomes. Environ Microbiol.

[b23] Hermans PW, van Soolingen D, Bik EM, de Haas PE, Dale JW, van Embden JD (1991). Insertion element IS987 from *Mycobacterium bovis* BCG is located in a hot-spot integration region for insertion elements in *Mycobacterium tuberculosis* complex strains. Infect Immun.

[b24] Horvath P, Romero DA, Coûté-Monvoisin A-C, Richards M, Deveau H, Moineau S (2008). Diversity, activity, and evolution of CRISPR loci in *Streptococcus thermophilus*. J Bacteriol.

[b25] Jore MM, Lundgren M, Van Duijn E, Bultema JB, Westra ER, Waghmare SP (2011). Structural basis for CRISPR RNA-guided DNA recognition by Cascade. Nat Struct Mol Biol.

[b26] Kamerbeek J, Schouls L, Kolk A, van Agterfeld M, van Soolingen D, Kuijper S (1997). Simultaneous detection and strain differentiation of *Mycobacterium tuberculosis* for diagnosis and epidemiology. J Clin Microbiol.

[b27] Lillestøl RK, Redder P, Garrett RA, Brügger K (2006). A putative viral defence mechanism in archaeal cells. Archaea.

[b28] Lillestøl RK, Shah SA, Brügger K, Redder P, Phan H, Christiansen J, Garrett RA (2009). CRISPR families of the crenarchaeal genius *Sulfolobus*: bidirectional transcription and dynamic properties. Mol Microbiol.

[b29] Lintner NG, Frankel KA, Tsutakawa SE, Alsbury DL, Copié V, Young MJ (2011). The structure of the CRISPR-associated protein Csa3 provides insight into the regulation of the CRISPR/Cas system. J Mol Biol.

[b30] Makarova KS, Haft DH, Barrangou R, Brouns SJ, Charpentier E, Horvath P (2011). Evolution and classification of the CRISPR-Cas systems. Nat Rev Microbiol.

[b31] Manica A, Zebec Z, Teichmann D, Schleper C (2011). *In vivo* activity of CRISPR-mediated virus defence in a hyperthermophilic archaeon. Mol Microbiol.

[b32] Marraffini LA, Sontheimer EJ (2008). CRISPR interference limits horizontal gene transfer in *Staphylococci* by targeting DNA. Science.

[b33] Mojica FJ, Diez-Villasenor C, Garcia-Martinez J, Soria E (2005). Intervening sequences of regularly spaced prokaryotic repeats derive from foreign genetic elements. J Mol Evol.

[b34] Mojica FJM, Díez-Villaseñor C, García-Martínez J, Almendros C (2009). Short motif sequences determine the targets of the prokaryotic CRISPR defence system. Microbiology.

[b35] Pina M, Bize A, Forterre P, Prangishvili D (2011). The archaeoviruses. FEMS Microbiol Rev.

[b36] Porter K, Russ BE, Dyall-Smith ML (2007). Virus–host interactions in salt lakes. Curr Opin Microbiol.

[b37] Pourcel C, Salvignol G, Vergnaud G (2005). CRISPR elements in *Yersinia pestis* acquire new repeats by preferential uptake of bacteriophage DNA, and provide additional tools for evolutionary studies. Microbiology.

[b38] Prangishvili D, Albers SV, Holz I, Arnold HP, Stedman K, Klein T (1998). Conjugation in archaea: frequent occurrence of conjugative plasmids in *Sulfolobus*. Plasmid.

[b39] Prangishvili D, Forterre P, Garrett RA (2006a). Viruses of the Archaea: a unifying view. Nat Rev Microbiol.

[b40] Prangishvili D, Vestergaard G, Häring M, Aramayo R, Basta T, Rachel R (2006b). Structural and genomic properties of the hyperthermophilic archaeal virus ATV with an extracellular stage of the reproductive cycle. J Mol Biol.

[b41] Rutherford K, Parkhill J, Crook J, Horsnell T, Rice P, Rajandream MA (2000). Artemis: sequence visualization and annotation. Bioinformatics.

[b43] Shah SA, Garrett RA (2011). CRISPR/Cas and Cmr modules, mobility and evolution of adaptive immune systems. Res Microbiol.

[b42] Shah SA, Hansen NR, Garrett RA (2009). Distributions of CRISPR spacer matches in viruses and plasmids of crenarchaeal acidothermophiles and implications for their inhibitory mechanism. Biochem Soc Trans.

[b44] Shah SA, Vestergaard G, Garrett RA, Marchfelder A, Hess W (2011). CRISPR/Cas and CRISPR/Cmr immune systems of Archaea. Regulatory RNAs in Prokaryotes.

[b45] She Q, Singh RK, Confalonieri F, Zivanovic Y, Allard G, Awayez MJ (2001). The complete genome of the crenarchaeon *Sulfolobus solfataricus* P2. Proc Natl Acad Sci USA.

[b46] Soler N, Marguet E, Verbavatz JM, Forterre P (2008). Virus-like vesicles and extracellular DNA from hyperthermophilic archaea of the order Thermococcales. Res Microbiol.

[b47] Swarts DC, Mosterd C, van Passel MWJ, Brouns SJJ (2012). CRISPR interference directs strand specific spacer acquisition. PLoS ONE.

[b48] Tang T-H, Bachellerie J-P, Rozhdestvensky T, Bortolin M-L, Huber H, Drungowski M (2002). Identification of 86 candidates for small non-messenger RNAs from the archaeon *Archaeoglobus fulgidus*. Proc Natl Acad Sci USA.

[b49] Tang T-H, Polacek N, Zywicki M, Huber H, Brügger K, Garrett R (2005). Identification of novel non-coding RNAs as potential antisense regulators in the archaeon *Sulfolobus solfataricus*. Mol Microbiol.

[b50] Tyson GW, Banfield JF (2008). Rapidly evolving CRISPRs implicated in acquired resistance of microorganisms to viruses. Environ Microbiol.

[b51] Wang Y, Duan Z, Zhu H, Guo X, Wang Z, Zhou J (2007). A novel *Sulfolobus* non-conjugative extrachromosomal element capable of integration into the host genome and spreading in the presence of a fusellovirus. Virology.

[b52] Wurtzel O, Sapra R, Chen F, Zhu YW, Simmons BA, Sorek R (2010). A single-base resolution map of an archaeal transcriptome. Genome Res.

[b53] Xiang X, Chen L, Huang X, Luo Y, She Q, Huang L (2005). *Sulfolobus tengchongensis* spindle-shaped virus STSV1: virus–host interactions and genomic features. J Virol.

[b54] Yosef I, Goren MG, Qimron U (2012). Proteins and DNA elements essential for the CRISPR adaptation process in *Escherichia coli*. Nucleic Acids Res.

[b55] Zhang J, Rouillon C, Kerou M, Reeks J, Brugger K, Graham S (2012). Structure and mechanism of the Cmr complex of CRISPR-mediated antiviral immunity. Mol Cell.

[b56] Zillig W, Kletzin A, Schleper C, Holz I, Janekovic D, Hain J (1994). Screening for Sulfolobales, their plasmids and their viruses in Icelandic solfataras. Syst Appl Microbiol.

[b57] Zillig W, Arnold HP, Holz I, Prangishvili D, Schweier A, Stedman K (1998). Genetic elements in the extremely thermophilic archaeon *Sulfolobus*. Extremophiles.

