# Supplementary material for: Selective and hyperactive uptake of foreign DNA by adaptive immune systems of an archaeon via two distinct mechanisms
Source: Mol Microbiol. 2012 Jul 27;85(6):1044–56. doi: 10.1111/j.1365-2958.2012.08171.x (PMC3468723; doi:10.1111/j.1365-2958.2012.08171.x)

Fig. S1. DNase I treatment of the purified virus mixture. PCR products derive from *orf174* within an operon of viral contig 1 (Table 3) and the *traG*-like gene in contig 2 of the putative conjugative plasmid (Table 4) amplified from isolated DNA before (-) and after (+) DNase I treatment.

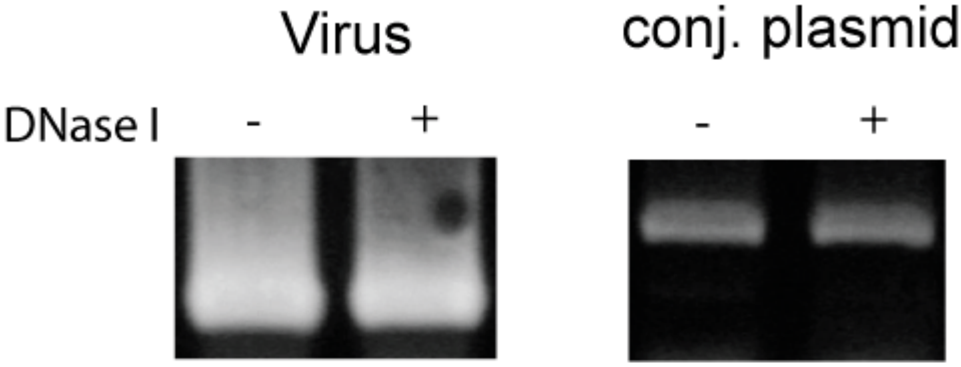

Fig. S2. Reversion of CRISPR loci A to D in the culture of the infected CRISPR-minus strain. PCR products amplified from leader proximal regions of CRISPR loci A to F. Template DNA has been isolated from 2 ml cells of the infected CRISPR-minus strain. Dpi - days post-infection.

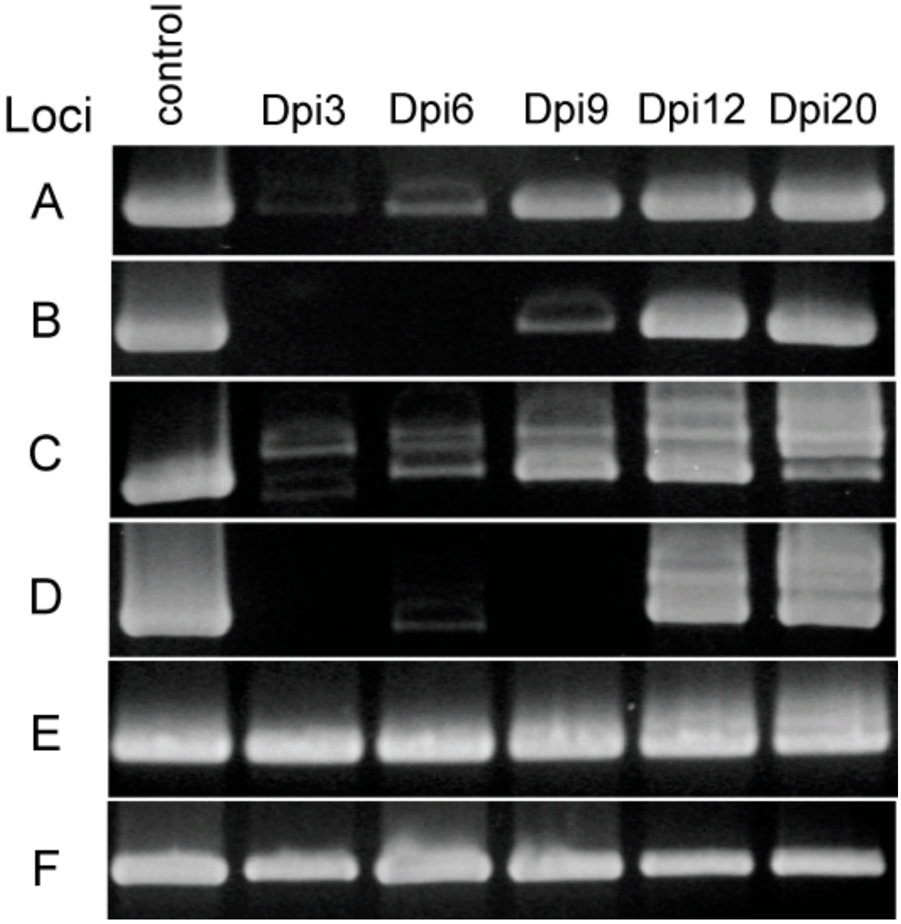

Supplement: Supplementary file 1 [file mmi0085-1044-SD1.pdf]
